# Second cancers and causes of death in patients with testicular cancer in Sweden

**DOI:** 10.1371/journal.pone.0214410

**Published:** 2019-03-28

**Authors:** Luyao Zhang, Otto Hemminki, Tianhui Chen, Hongyao Yu, Guoqiao Zheng, Subhayan Chattopadhyay, Asta Försti, Kristina Sundquist, Jan Sundquist, Kari Hemminki

**Affiliations:** 1 Division of Molecular Genetic Epidemiology, German Cancer Research Center (DKFZ), Heidelberg, Germany; 2 Faculty of Medicine, University of Heidelberg, Heidelberg, Germany; 3 Department of Urology, Helsinki University Hospital, Helsinki, Finland; 4 Cancer Gene Therapy Group, Faculty of Medicine, University of Helsinki, Helsinki, Finland; 5 Group of Molecular Epidemiology and Cancer Precision Prevention, Zhejiang Academy of Medical Sciences (ZJAMS), Hangzhou, China; 6 Center for Primary Health Care Research, Lund University, Malmö, Sweden; 7 Department of Family Medicine and Community Health, Department of Population Health Science and Policy, Icahn School of Medicine at Mount Sinai, New York, United States of America; 8 Center for Community-based Healthcare Research and Education (CoHRE), Department of Functional Pathology, School of Medicine, Shimane University, Shimane, Japan; National Health Research Institutes, TAIWAN

## Abstract

While treatment for testicular cancer (TC) has become standardized after the 1980s with an associated significant improvement in patient survival, this has been accompanied by an increased risk of second primary cancers (SPCs). Patients were identified from the Swedish Cancer Registry spanning the years from 1980 to 2015, including 8788 individuals with primary TC and their SPCs. Relative risks (RRs) for SPC were calculated using the generalized Poisson regression model. SPCs were diagnosed in 9.4% of patients with TC and half of them were late onset cancers not common in the population in their 40s. Overall RR of SPCs (excluding second TC) was 1.30 (95%CI: 1.20–1.40), including high risks for seven solid cancers, non-Hodgkin lymphoma and leukemia. Second TC was the most common SPC and the RR of 17.19 (95%CI: 14.89–19.85) was the highest recorded. Cancers known to be fatal as first primary cancers were also fatal as SPC in TC patients. Survival at 30 years of follow-up was approximately 80% for TC patients without SPC but it decreased to 40% for patients with SPC. The unexpected finding that half of the identified SPCs were typical late onset cancers in the middle-aged population raises concerns that therapy may facilitate premature aging. The risks of SPC are clinically important for the long-term management of TC patients and the high-mortality calls for a future management strategy.

## Introduction

Testicular cancers (TCs) are predominantly germ cell tumors of two main types, seminomas and non-seminomas, the latter including teratoma and embryonal tumor as the largest histological types [1]. The incidence of testicular cancer has increased in many countries, and it is highest in developed countries and lowest in developing countries [2]. In Europe, the incidence of TC has dramatically increased with an equally dramatic decrease in mortality [3, 4]. The 5-year relative survival in Europe and USA around the year 2005 was close to 99% for seminoma and 94% for non-seminoma [5]. Aberrant fetal gonocytes are precursor lesions of TC constituting in-situ lesions, which gain invasive phenotype usually in early adulthood [6, 7]. Individual risk factors of TC include genital malformations such as cryptorchidism, hypospadias and inguinal hernia; in addition, subfertility and some birth-related factors have also been associated with TC [8–10].

The dramatic decrease in mortality concerning TC is one of the success stories of cancer treatment [11]. It was achieved through several discoveries, including therapeutic effects of cisplatin in the1960s and adoption of BEP (bleomycin, etoposide and cisplatin) regimen in the 1980s [11]. The treatment depends on whether the disease is localized or metastatic, including orchiectomy and BEP and, in addition, radiation for seminoma that is radiosensitive [12]. Radiation and chemotherapy agents in BEP are DNA-damaging and concerns of second primary cancers (SPCs) have been relevant [13]. It was estimated that the risk of SPCs increases 2.6-fold after subdiaphragmatic radiotherapy and 2.1-fold after chemotherapy when the median follow-up time was 17.6 years [14]. This was in-line with a more recent study with a median follow-up of 14.4 years; more than one line of treatments increased the risk of SPCs to 3.7 [15]. According to a qualitative risk estimation conducted by the International Agency for Research on Cancer ‘there is sufficient evidence in humans for the carcinogenicity of etoposide in combination with cisplatin and bleomycin. Etoposide in combination with cisplatin and bleomycin causes acute myeloid leukaemia’ [16]. In view of the known relative short ‘latency time’ for acute myeloid leukemia compared to solid tumors, it is relevant to consider possible risks for even solid tumors in TC patients when follow-up times are becoming longer [17, 18]. A recent study reported a 25-year cumulative incidence of solid SPC at 10.3% [19]. In nonseminoma patients increased risks were noted for SPCs of the thyroid, lung, stomach, pancreas, colon, and bladder and of melanoma and soft tissue sarcoma while the risks among seminoma patients covered the small intestine, pancreas, and urinary bladder.

We conducted here a nationwide Swedish study on survivors of TC diagnosed after 1980 when the successful therapies were introduced. Causes of death were considered in patients with and without SPCs. This is an extension of our study published in 2010 focusing only on the risk of SPC with a sample size of 40% of the present numbers [20]. Even if we have no data on the therapies applied, which is a weakness compared to some previous studies, we are able to show novel features of SPC and the related mortality [21–23].

## Subjects and methods

The Swedish Family-Cancer Database was used to estimate the familial relative risks of the major identifiable malignancies in the Swedish population. This database was created by linking information from the Multi-Generation Register, national censuses, Swedish Cancer Registry and death notifications [24]. The linkages were conducted at the National Board for Health and Welfare using the personal identifiers, which were removed before delivery of the datasets to the users. The 2015 update of these data includes a total population of over 16.1 million individuals. The Swedish Cancer Registry requires the compulsory reporting of all diagnosed malignancies and provides more than 90% coverage of all cancer diagnoses in Sweden [25]. The registry relies on separate compulsory notifications from clinicians and pathologists/cytologists who diagnosed the neoplasms [26]. The registry counts tumors, not patients, except for skin and urinary tract tumors diagnosed at the same topological area (https://www.ancr.nu/dyn/resources/File/file/7/4247/1412940269/total_document_survey_optimeret.pdf). To classify cancer types, the Swedish Cancer Register has used the following International Classification of Disease codes: (ICD)-7 since 1958, ICD-9 since 1987, SNOMED (ICD-O/2) since 1993, and ICD-O/3 since 2005. The degree of histological verification of TC has been close to 100% [27]. In addition to seminoma and non-seminoma, some rare undefined histologies (<2% of all cases) were not included in the histology-specific analyses; this was the only exclusion criteria applied and the reason why the total number to patients was somewhat higher than the sum of patients with seminoma and nonseminoma. Laterality of TC was not available. An *ad hoc* study on the diagnostic accuracy of second neoplasms in the Swedish Cancer Registry found 98% to be correctly classified; no recorded SPC was found to be a metastasis upon reexamination [28].

Relative risks (RRs) were assessed with incidence rate ratios, regressed over a fixed effects generalized Poisson model. RRs for SPC were obtained by comparing incidence rates for SPC X in TC patients with rates for first cancer X in the background population. Follow-up was started at diagnosis of TC after January 1, 1980 and terminated at diagnosis of SPC, emigration, death or December 31, 2015, whichever occurred first. Identical follow-up times were applied when TC was considered as SPC. Sex (for cancers in both sexes), age group, calendar-period, socioeconomic status and residential areas were treated as potential confounders and were adjusted for in the regression model. Confidence intervals (CIs) were calculated for 5% level of significance. For survival analysis multivariate Cox-proportional hazard regression was used (adjustments as above) and Kaplan-Meier curves were generated to depict survival probabilities stratified over SPC diagnosis.

All cancer-related deaths were stratified into TC, SPC, higher order primaries, ‘other cancer’ and non-neoplastic cause of death. Higher order primaries were 3nd, 4^th^ etc primary cancers. ‘Other cancer’ includes cases diagnosed at the issue of death certificates, referred to as ‘death certificate notifications’ [26]. These death notifications are not used by the Swedish Cancer Registry to complement cancer data, opposite to the other Nordic Cancer Registries [25, 26]. We have found that the notifications often included multiple cancers and cancer of unknown primary (CUP). As CUP is a metastatic cancer, even other listed cancers, if not those reported to the Cancer Registry, may be metastases [29]. If the death certificate notification matched the organ site of the reported primary cancer it was classified to that site but in most cases such a reclassification could not be made and the classification was to ‘other neoplasia’.

All the above statistical analyses were done with SAS version 9.4.

The study was approved by the Ethical Review Board of Lund University (Reg.nr 2012/795), Sweden and conducted in accordance with the tenets of the Declaration of Helsinki.

## Results

The Family-Cancer Database included 8788 TCs of which 4879 were seminomas and 3717 non-seminomas diagnosed since the year 1980 (Table 1). The respective number of SPCs after these cancers were 830 (9.4%), 568 (11.6%) and 249 (6.7%), for which the median follow-up times were 11, 11 and 13 years respectively.

**Table 1:** The number and age at diagnosis of patients with testicular cancer and number of patients with second primary cancer.

**Testicular cancer histology**	**N (%)**	**Median age at diagnosis IQR**^**1**^**(years)**
Testicular cancer	8788 (100.0)	34 (27–42)
Seminoma	4879 (55.5)	38 (31–45)
Non-seminoma	3717 (42.3)	29 (24–36)
**SPC**^**2**^**s following testicular cancer**	**N (%)**	**Median follow-up time IQR (years)**
Testicular cancer	830 (9.4)	11 (4–19)
Seminoma	568 (11.6)	11 (4–19)
Non-seminoma	249 (6.6)	13 (4–20)

IQR^1^ = Interquartile range (lower quartile-upper quartile)

SPC^2^ = second primary cancer

Risks for SPCs are shown in Table 2 after TCs. Cancers are listed when at least three SPCs were diagnosed after TC. The most common SPCs were TC and prostate cancer. The RR for second TC was 17.19 (95%CI: 14.89–19.85), and 2.1% of TC patients were afflicted with a second TC. Overall RR for SPC (excluding TC) after TC was 1.30 (95%CI: 1.20–1.40). Risks for a total of nine non-TC SPCs were increased; the highest increases being for thyroid gland (2.64, 95%CI: 1.32–5.29) and connective tissue (2.60, 95%CI: 1.40–4.84) cancer. Some typically old age cancers were common as SPCs, prostate cancer (1.16, 95%CI: 1.00–1.36) accounting for 1/4 of all cases (the median age of onset for prostate cancer was 64 years). Others were stomach, esophageal, liver, bladder and skin (squamous cell) cancers, which combined accounted for another 1/4 of all cases. RRs were over 1.00 for all of them, and these were significant for prostate (1.16, 95%CI: 1.00–1.36), bladder (1.78, 95%CI: 1.36–2.33) and skin (1.52, 95%CI: 1.07–2.15) cancers. The RR for these six cancers combined was 1.35 (N = 283, 95%CI: 1.21–1.49). For subtypes of leukemia, risks for acute and chronic myeloid leukemia and acute lymphoid leukemia were increased (all RRs over 3.00).

**Table 2:** RRs of second primary cancers in survivors of testicular cancer.

Second cancer site	After testicular cancer		
	Cases	RR^1^	95%CI^2^
All (excluding testis)	639	**1.30**	**1.20–1.40**
Upper aerodigestive tract	12	0.78	0.45–1.38
Esophagus	9	1.58	0.82–3.03
Stomach	16	1.40	0.86–2.29
Small intestine	3	1.15	0.37–3.58
Colorectum	67	**1.33**	**1.05–1.69**
Liver	12	1.25	0.71–2.21
Pancreas	14	1.40	0.83–2.36
Lung	36	0.98	0.71–1.36
Prostate	161	**1.16**	**1.00–1.36**
Testis	191	**17.19**	**14.89–19.85**
Other male genital	4	2.11	0.79–5.63
Kidney	32	**2.19**	**1.55–3.10**
Urinary bladder	53	**1.78**	**1.36–2.33**
Melanoma	33	1.13	0.80–1.58
Skin, squamous cell	32	**1.52**	**1.07–2.15**
Eye	3	1.93	0.62–6.00
Nervous system	24	1.37	0.92–2.05
Thyroid gland	8	**2.64**	**1.32–5.29**
Endocrine glands	8	1.10	0.55–2.19
Connective tissue	10	**2.60**	**1.40–4.84**
Non-Hodgkin lymphoma	36	**1.87**	**1.35–2.59**
Hodgkin lymphoma	3	0.98	0.32–3.04
Myeloma	9	1.36	0.71–2.62
Leukemia	31	**1.99**	**1.40–2.83**
Acute myeloid leukemia	10	**3.59**	**1.93–6.67**
Chronic myeloid leukemia	5	**3.07**	**1.27–7.39**
Acute lymphoid leukemia	3	**3.58**	**1.15–11.13**
Chronic lymphoid leukemia	7	1.29	0.62–2.71
Others	6	1.54	0.69–3.42
CUP^3^	18	1.55	0.98–2.47

RR^1^ = relative risk, 95%CI^2^ = 95% confidence interval, bold font indicates that the lower limit of 95%CI does not include 1.00. CUP^3^ = cancer of unknown primary.

In Table 3 data were analyzed by follow-up of less than five years or more than five years; the overall RRs were 1.52 (95%CI: 1.26–1.83) and 1.55 (95%CI: 1.42–1.68). Risks for colorectal, bladder and connective tissue cancers and non-Hodgkin lymphoma were only increased in the long follow-up period, while risks for other male genital, kidney, skin (squamous cell) and thyroid cancers, myeloma and CUP were only increased in the first period. Leukemia risk was increased in both periods, more in the first period (3.65 vs 1.73) when the RR for acute myeloid leukemia reached 6.56 (95%CI: 2.46–17.50).

**Table 3:** Relative risks (RRs) of second primary cancers in survivors of testicular cancer by follow-up time.

Second cancer site	<5 yrs.			> = 5 yrs.		
	Cases	RR^1^	95%CI^2^	Cases	RR	95%CI
All (excluding testis)	109	**1.52**	**1.26–1.83**	530	**1.55**	**1.42–1.68**
Upper aerodigestive tract	0	--	--	12	0.94	0.54–1.66
Esophagus	1	1.32	0.19–9.39	8	1.65	0.82–3.30
Stomach	3	1.21	0.39–3.76	13	1.42	0.83–2.45
Small intestine	0	--	--	13	1.39	0.45–4.32
Colorectum	3	0.33	0.11–1.04	64	**1.51**	**1.18–1.93**
Liver	0	--	--	12	1.52	0.86–2.68
Pancreas	3	1.60	0.52–4.97	11	1.32	0.73–2.39
Lung	4	0.60	0.23–1.60	32	1.04	0.74–1.48
Prostate	24	1.22	0.82–1.82	137	1.13	0.96–1.34
Testis	111	**25.51**	**21.15–30.77**	80	**11.86**	**9.51–14.79**
Other male genital	2	**7.12**	**1.78–28.50**	2	1.27	0.32–5.09
Kidney	16	**5.70**	**3.49–9.31**	16	1.32	0.81–2.16
Urinary bladder	5	0.96	0.40–2.32	48	**1.92**	**1.44–2.54**
Melanoma	6	1.01	0.45–2.24	27	1.13	0.77–1.65
Skin, squamous cell	6	**2.24**	**1.01–4.99**	26	1.45	0.99–2.13
Eye	0	--	--	3	2.36	0.76–7.34
Nervous system	7	1.63	0.78–3.42	17	1.26	0.78–2.03
Thyroid gland	4	**5.14**	**1.93–13.70**	4	1.73	0.65–4.62
Endocrine glands	3	1.81	0.58–5.62	5	0.87	0.36–2.08
Connective tissue	1	1.42	0.20–10.05	9	**2.99**	**1.55–5.75**
Non-Hodgkin lymphoma	7	1.84	0.88–3.86	29	**1.83**	**1.27–2.64**
Hodgkin lymphoma	0	--	--	3	1.45	0.47–4.50
Myeloma	3	**3.23**	**1.04–10.02**	6	1.08	0.49–2.41
Leukemia	9	**3.65**	**1.90–7.02**	22	**1.73**	**1.14–2.63**
Acute myeloid leukemia	4	**6.56**	**2.46–17.50**	6	**2.76**	**1.24–6.14**
Chronic myeloid leukemia	1	2.30	0.32–16.38	4	**3.35**	**1.26–8.95**
Acute lymphoid leukemia	0	--	--	3	**5.23**	**1.68–16.28**
Chronic lymphoid leukemia	1	1.11	0.16–7.86	6	1.33	0.60–2.97
Others	3	**4.14**	**1.34–12.85**	3	0.94	0.30–2.93
CUP^3^	1	0.45	0.06–3.17	17	**1.78**	**1.11–2.87**

RR^1^ = relative risk, 95%CI^2^ = 95% confidence interval, bold font indicates that the lower limit of 95%CI does not include 1.00, CUP^3^ = cancer of unknown primary.

SPC risk was higher after non-seminoma (1.43, 95%CI: 1.24–1.66) than after seminoma (1.28, 95%CI: 1.16–1.40) (Table 4). For second TC the order was reversed, RR being higher after seminoma (23.30, 95%CI: 19.49–27.86) compared to non-seminoma (11.61, 95%CI: 9.11–14.80, note non-overlapping 95%CIs); second TC was diagnosed in 2.5% of seminoma patients and in 1.8% of non-seminoma patients. While risks for kidney, bladder and connective tissue cancers and leukemia were high in both types of TC, esophageal and thyroid cancers and non-Hodgkin lymphoma were in excess only after seminoma and colorectal cancer only after non-seminoma. However, because of the small case numbers the 95% CIs were wide and overlapping.

**Table 4:** RRs of second primary cancers after seminoma and non-seminoma.

Second cancer site	Seminoma			Non-seminoma		
	Cases	RR^1^	95%CI^2^	Cases	RR	95%CI
All (excluding testis)	445	**1.28**	**1.16–1.40**	183	**1.43**	**1.24–1.66**
Upper aerodigestive tract	8	0.75	0.37–1.50	4	0.89	0.34–2.38
Esophagus	9	**2.17**	**1.13–4.18**	0	--	--
Stomach	14	1.67	0.99–2.82	2	0.72	0.18–2.88
Colorectum	46	1.26	0.94–1.68	21	**1.64**	**1.07–2.51**
Liver	9	1.30	0.67–2.49	3	1.22	0.39–3.78
Pancreas	12	1.64	0.93–2.89	2	0.79	0.20–3.16
Lung	21	0.79	0.51–1.21	15	1.62	0.98–2.69
Prostate	115	1.11	0.92–1.33	38	1.22	0.88–1.67
Testis	123	**23.30**	**19.49–27.86**	66	**11.61**	**9.11–14.80**
Kidney	23	**2.22**	**1.48–3.34**	9	**2.20**	**1.15–4.23**
Urinary bladder	34	**1.57**	**1.12–2.19**	19	**2.57**	**1.64–4.03**
Melanoma	22	1.15	0.76–1.74	9	1.10	0.61–1.98
Skin, squamous cell	21	1.35	0.88–2.08	11	1.87	0.97–3.59
Eye	3	2.87	0.92–8.90	0	--	--
Nervous system	17	1.53	0.95–2.46	7	1.12	0.53–2.34
Thyroid gland	6	**3.19**	**1.43–7.10**	2	1.75	0.44–7.01
Endocrine glands	7	1.48	0.70–3.10	0	**--**	--
Connective tissue	6	**2.39**	**1.07–5.32**	4	**3.08**	**1.16–8.23**
Non-Hodgkin lymphoma	26	**1.96**	**1.33–2.88**	10	1.73	0.93–3.21
Myeloma	5	1.05	0.44–2.51	4	2.36	0.89–6.29
Leukemia	21	**1.96**	**1.28–3.00**	10	**2.16**	**1.16–4.02**
Acute myeloid leukemia	8	**4.27**	**2.13–8.55**	2	2.42	0.60–9.68
Chronic myeloid leukemia	2	2.01	0.50–8.06	3	**5.01**	**1.61–15.56**
Acute lymphoid leukemia	2	**4.42**	**1.10–17.71**	1	2.73	0.38–19.38
Chronic lymphoid leukemia	6	1.55	0.69–3.45	1	0.75	0.10–5.29
Others	3	1.14	0.37–3.53	3	2.62	0.84–8.12
CUP^3^	12	1.44	0.82–2.53	6	1.99	0.89–4.43

RR^1^ = relative risk, 95%CI^2^ = 95% confidence interval, bold font indicates that the lower limit of 95%CI does not include 1.00. CUP^3^ = cancer of unknown primary.

In TC without SPC, non-neoplastic causes were the most common cause of death (52.3%) and TC ranked the second (39.4%) (Table 5). However, these percentages were reversed in seminoma (71.2% vs. 21.6%) compared to non-seminoma (33.9% vs. 59.2%), (*P*<0.0001). In TC with SPC, non-TC SPCs were the main cause of death overall (52.3%), in seminoma (52.2%) as well as in non-seminoma (54.2%). Non-neoplastic causes ranked second (23.5% in all TC), followed by other cancers (13.2%), higher order primaries (i.e., 3nd, 4^th^ etc primary cancers, 6.3%) and TC (first or SPC combined, 4.6%). ‘Other cancers’ were reported in death certificates but had not been reported to the Cancer Registry (see Methods).

**Table 5:** Cause of death in testicular cancer with or without second primary cancer.

**Cause of death**		**Without second primary cancer**		
		**Testicular cancer (N %)**	**Seminoma (N %)**	**Non-seminoma (N %)**
Testicular cancer		291 (39.4)	80 (21.6)	173 (59.2)
Other cancers		61 (8.3)	27 (10.3)	20 (6.8)
Non-neoplastic		386 (52.3)	264 (71.2)	99 (33.9)
All		738 (100.0)	371 (100.0)	292 (100.0)
**Cause of death**		**With second primary cancer**		
		**Testicular cancer (N %)**	**Seminoma (N %)**	**Non-seminoma (N %)**
Testicular cancer^1^		14 (4.6)	8 (3.5)	6 (8.3)
SPC^2^	non-testicular cancer	158 (52.3)	118 (52.2)	39 (54.2)
Higher order primary		19 (6.3)	15 (6.6)	4 (5.6)
Other cancers		40 (13.2)	28 (12.4)	11 (15.3)
Non-neoplastic		71 (23.5)	57 (25.2)	12 (16.7)
All		302 (100.0)	226 (100.0)	72 (100.0)

TC^1^ = testicular cancer, including first primary cancer and second primary cancer. SPC^2^ = second primary cancer.

Causes of death in TC patients are listed according to the type of SPC (Table 6). Sites were included when at least four deaths occurred. When the SPC was a known fatal cancer, such as pancreatic, esophageal and liver cancer, all patients died because of the SPC. Even for colorectal and lung cancers as SPC the cause of death was SPC in more than 80% of cases. No patient died of skin cancer when this was the SPC.

**Table 6:** Causes of death in patients diagnosed with second primary cancer after testicular cancer.

Second primary cancer	Total number of deaths	Cause of death N (%)				
		TC^1^	SPC^2^	HOPC^3^	OC^4^	Non-neoplastic
Upper aerodigestive tract	9	0	5 (55.6)	0	3 (33.3)	1 (11.1)
Esophagus	8	0	8 (100.0)	0	0	0
Stomach	14	0	10 (71.4)	0	2 (14.3)	2 (14.3)
Colorectum	30	1 (3.3)	26 (86.7)	0	1 (3.3)	2 (6.7)
Liver	7	0	7 (100.0)	0	0	0
Pancreas	13	0	13 (100.0)	0	0	0
Lung	28	1(3.6)	23 (82.1)	1 (3.6)	3 (10.7)	0
Prostate	47	4 (8.5)	17 (36.2)	5 (10.6)	3 (6.4)	18 (38.3)
Testis	11	5 (45.5)	0	2 (18.2)	1 (9.1)	3 (27.3)
Kidney	12	0	5 (41.7)	1 (8.3)	1 (8.3)	5 (41.7)
Urinary bladder	21	1 (4.8)	9 (42.9)	5 (23.8)	1 (4.8)	5 (23.8)
Melanoma	8	0	3 (37.5)	1 (12.5)	0	4 (50.0)
Skin, squamous cell	12	0	0	0	1 (8.3)	11 (91.7)
Nervous system	12	1 (8.3)	8 (66.7)	0	1 (8.3)	2 (16.7)
Thyroid gland	4	0	1 (25.0)	0	1 (25.0)	2 (50.0)
Connective tissue	6	0	0	0	5 (83.3)	1 (16.7)
Non-Hodgkin lymphoma	13	0	4 (30.8)	0	4 (30.8)	5 (38.5)
Myeloma	5	0	4 (80.0)	0	0	1 (20.0)
Leukemia	15	0	11 (73.3)	2 (13.3)	1 (6.7)	1 (6.7)
Acute myeloid leukemia	6	0	5 (83.3)	0	1 (16.7)	0
Chronic myeloid leukemia	1	0	1 (100.0)	0	0	0
Acute lymphoid leukemia	2	0	1 (50.0)	1 (50.0)	0	0
Chronic lymphoid leukemia	2	0	1 (50.0)	1 (50.0)	0	0
CUP^5^	17	1 (5.9)	3 (17.6)	0	9 (52.9)	4 (23.5)
All	302	14 (4.6)	158 (52.3)	19 (6.3)	40 (13.2)	71 (23.5)

TC^1^ = testicular cancer, included first primary cancer and second primary cancer.

SPC^2^ = second primary cancer. HOPC^3^ = higher (3th, 4th) order primary cancer.

OC^4^ = other cancer (excluding second primary cancer and higher order primary cancer and testicular cancer).

CUP^5^ = cancer of unknown primary.

**Fig 1:**
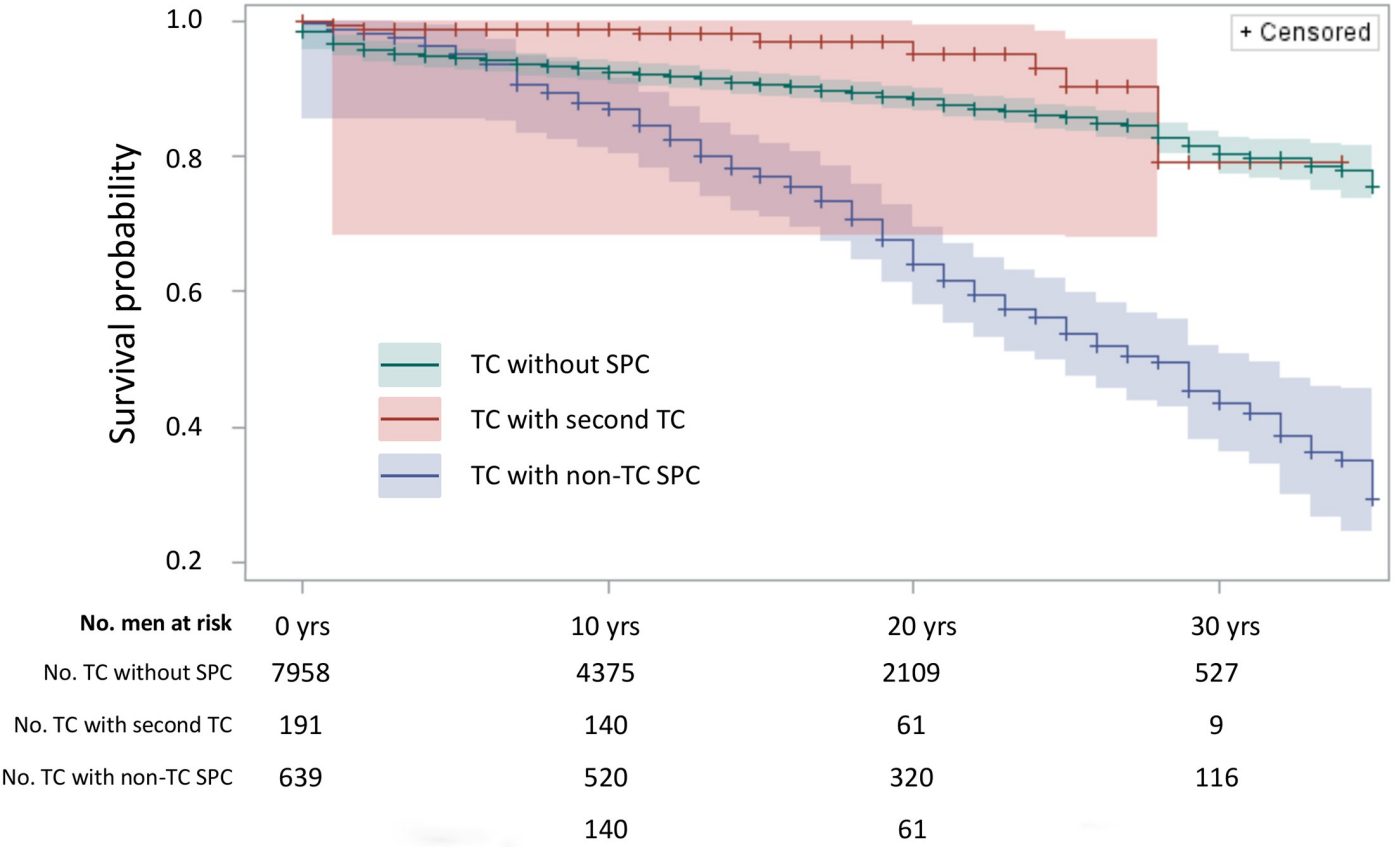
Follow-up time since diagnosis of testicular cancer (years). Survival probability of TC patients without SPC, TC patients with TC as SPC and TC patients with SPC other than TC. Shading around the plots show 95%CIs. Men at risk at each time point are listed below the figure.

The survival probabilities for TC patients are shown in Fig 1, including TC without SPC, TC with TC as SPC, and TC with SPC but excluding patients with TC as SPC. Survival was similar in the first 10 years after diagnosis of TC because SPCs were diagnosed in the course of time (median time to SPC 11y, Table 1). Later survival curves diverged with marked poorer survival for patients with SPC other than TC compared to those without SPC. At 30 years of follow-up about 80% of TC patients without SPC were alive, compared to about 40% for patients with SPC. The top curve for patients who had TC as SPC was based on only 11 deaths. The hazard ratio, adjusted for a number of variables, was 1.45 (95%CI: 1.25–1.67) for patients with SPC other than TC compared to those without SPC.

## Discussion

The present results on SPCs provide novel insight into possible differential development of SPC after seminoma and non-seminoma, and on mortality related to SPC. While 9.4% of TC patients developed a SPC, the percentage was higher in seminoma (11.6%) compared to non-seminoma (6.7%), (*P*<0.0001) but the risk of SPC was marginally higher for non-seminoma compared to seminoma. As the median diagnostic age of TC was 34 years and the median follow-up time was 11 years, a large proportion of patients were diagnosed with SPC before age 50 years. It is curious that the diagnosed SPCs, with the exception of second TC, were not typically mid-age cancers. Prostate cancer and five other typically late onset cancers accounted for half of all SPCs after TC; their combined RR was 1.35 (95%CI: 1.21–1.49). Signs of early aging in the form of ‘physiologic frailty’ have been described previously for childhood cancer survivors [30, 31]. This was defined as a combination of low muscle mass, self-reported exhaustion, low energy expenditure, slow walking speed, and weakness; this physiologic frailty was associated with risk of death. In childhood leukemia survivors, evidence of cellular aging was shown by leukocyte telomere length measurements which matched lengths for 20 years older healthy individuals [32]. Many signs of physiologic aging have been also recorded for TC patients [21].

The current therapeutic principles for treatment of TC were adopted in the 1980s. The maximal follow-up times of the surviving patients now exceed three decades and it will be possible to assess the full scope of SPCs in TC survivors, as reviewed [11, 21]. Evidence of an increased risk of leukemias was observed first and it was also seen in the present study for all leukemia and acute and chronic myeloid leukemia and acute lymphoid leukemia [23, 33]. Risks for all leukemia and acute myeloid leukemia were higher in the long follow-up period (5+ years) while risks for chronic myeloid leukemia and acute lymphoid leukemia were confined to the early follow-up period. A group of experts concluded after reviewing the available data on SPCs after TC that among solid tumors significantly increased risks were demonstrated, three- to seven-fold risks of cancers of the kidney, thyroid, and soft tissue [21]. The conclusion was based on patients receiving chemotherapy and who were compared to patients who underwent surgery alone [22]. Patients in our cohort also received radiotherapy and the risk for the above cancers were increased along with five other non-TCs. RRs for kidney and thyroid cancers were increased in the long follow-up period.

According to the literature, approximately 5% of men with TC run a risk of contralateral TC, of which 1/3 are synchronous tumors, and 2/3 metachronous tumors [34]. However, lower percentages of 2.1 to 2.5% have been found in other studies on unknown laterality [20]. The present figure was 2.1%, higher for seminoma (2.5%) than for non-seminoma (1.8%). The RRs were also higher (23.30, 95%CI: 19.49–27.86) for seminoma compared to non-seminoma (11.61, 95%CI: 9.11–14.80). The risk for second TC was higher (25.51, 95%CI: 21.15–30.77) in short (5+ year) follow-up compared to long follow-up (11.86, 95%CI: 9.51–14.79). The reasons for high contralateral TC extending throughout the follow-up period are likely to be related to common developmental history and risk factors. This is consistent with data on precursor lesions of TC which arise and accumulate following aberrant fetal gonocyte development and which may occur in both testicles [6, 7]. However, as the risks of second TCs appeared to increase with follow-up time, interacting therapeutic and developmental damage may drive such long-term risks. Nevertheless, such high risks warrant alertness in the patient follow-up, even if the limited current data showed no survival disadvantage for patients with TC as SPC.

In the survival analysis, we showed that at 30 years of follow-up about 80% of TC patients without SPC were alive, compared to about 40% for patients with SPC. The most common causes of death in TC patients without SPC were non-neoplastic causes claiming 52.3% of deaths, compared to 39.4% for TC. The pattern for non-neoplastic causes was even stronger in seminoma whereas in non-seminoma TC was the main cause of death. In TC with SPC, SPCs were the main cause of death overall (52.3%), as well as in seminoma as in non-seminoma. The type of SPCs influenced the causes of death such as patients with fatal cancer of the pancreas, esophagus and liver; all patients died of SPC. A Norwegian study on long-term survival in TC patients reported excellent survival in the first decade followed by ‘accelerated decline’ during 15 to 30 years of follow-up which they ascribed to late effects of treatment without distinguishing SPCs [35].

Lack of data on treatment is the major limitation of the study. Even though the main treatment guidelines have been standardized our inability to distinguish the group of patients undergoing surgery only excludes the possibility for internal comparisons. Another limitation is the late introduction of the TNM classification in the Cancer Registry at around 2003. As a consequence, we could identify only less than 100 TC patients with SPC and stage data; for half of them the SPC was TC. TNM classification is not extended to SPCs which does not allow assessment of the possible lead time bias in SPC diagnosis.

In summary, during the median follow-up time of 11 to 13 years close to 10% of TC patients were subsequently diagnosed with SPC. This had unfavorable prognostic consequences, particularly if the SPC was known to be a fatal first cancer. Curiously, about half of the identified SPCs were typical late-onset cancers, which suggests that therapy had facilitated premature aging. While therapy is likely to contribute to the risk of SPCs, the very high risks for second TC likely have primarily embryonic origins. Second TCs were diagnosed in 2.1% of the patients and the risks appeared to increase over follow-up time calling for a long-term management plan for TC patients.
